# The Potential Regimen of Target-Controlled Infusion of Propofol in Flexible Bronchoscopy Sedation: A Randomized Controlled Trial

**DOI:** 10.1371/journal.pone.0062744

**Published:** 2013-04-24

**Authors:** Ting-Yu Lin, Yu-Lun Lo, Chung-Hsing Hsieh, Yung-Lun Ni, Tsai-Yu Wang, Horng-Chyuan Lin, Chun-Hua Wang, Chih-Teng Yu, Han-Pin Kuo

**Affiliations:** Department of Thoracic Medicine, Chang Gung Memorial Hospital, College of Medicine, Chang Gung University, Taipei, Taiwan; Università Vita-Salute San Raffaele, Italy

## Abstract

**Objectives:**

Target-controlled infusion (TCI) provides precise pharmacokinetic control of propofol concentration in the effect-site (Ce), eg. brain. This pilot study aims to evaluate the feasibility and optimal TCI regimen for flexible bronchoscopy (FB) sedation.

**Methods:**

After alfentanil bolus, initial induction Ce of propofol was targeted at 2 μg/ml. Patients were randomized into three titration groups (i.e., by 0.5, 0.2 and 0.1 μg/ml, respectively) to maintain stable sedation levels and vital signs. Adverse events, frequency of adjustments, drug doses, and induction and recovery times were recorded.

**Results:**

The study was closed early due to significantly severe hypoxemia events (oxyhemoglobin saturation <70%) in the group titrated at 0.5 μg/ml. Forty-nine, 49 and 46 patients were enrolled into the 3 respective groups before study closure. The proportion of patients with hypoxemia events differed significantly between groups (67.3 vs. 46.9 vs. 41.3%, *p* = 0.027). Hypotension events, induction and recovery time and propofol doses were not different. The Ce of induction differed significantly between groups (2.4±0.5 vs. 2.1±0.4 vs. 2.1±0.3 μg/ml, *p* = 0.005) and the Ce of procedures was higher at 0.5 μg/ml titration (2.4±0.5 vs. 2.1±0.4 vs. 2.2±0.3 μg/ml, *p* = 0.006). The adjustment frequency tended to be higher for titration at 0.1 μg/ml but was not statistically significant (2 (0∼6) vs. 3 (0∼6) vs. 3 (0∼11)). Subgroup analysis revealed 14% of all patients required no further adjustment during the whole sedation. Comparing patients requiring at least one adjustment with those who did not, they were observed to have a shorter induction time (87.6±34.9 vs. 226.9±147.9 sec, p<0.001), a smaller induction dose and Ce (32.5±4.1 vs. 56.8±22.7 mg, p<0.001; 1.76±0.17 vs. 2.28 ±0.41, p<0.001, respectively), and less hypoxemia and hypotension (15.8 vs.56.9%, p = 0.001; 0 vs. 24.1%, p = 0.008, respectively).

**Conclusion:**

Titration at 0.5 μg/ml is risky for FB sedation. A subgroup of patients required no more TCI adjustment with fewer complications. Further studies are warranted to determine the optimal regimen of TCI for FB sedation.

**Trial Registration:**

ClinicalTrials.gov NCT01101477

## Introduction

Propofol (2,6-diisopropylphenol) has been reported to be an ideal agent for flexible bronchoscopy (FB) sedation because of its fast effect and rapid recovery profile [1], [2], [3], [4]. However, controversy persists because of individualised patient responsiveness to propofol and the easy shift to deeper levels of sedation which has an associated risk of cardiopulmonary depression [5]. Propofol titration is most often based on the physician's discretion without fully taking into account the individual pharmacokinetic (PK) differences [1], [2], [3]. This may result in unstable drug plasma concentrations, fluctuant sedative levels as well as increasing cardio-respiration suppression [6], [7].

Target-controlled infusion (TCI), based on three-compartment models of propofol [8], could give precise PK control. This computer-assisted infusion algorithm integrates individual variables and then provides an infusion profile to achieve a steady plasma concentration [9], [10] or “effect-site” concentration (Ce) [6], [11], the theoretical drug concentration in the brain, and avoids unusual drug fluctuations. A steady-state sedation level could be maintained that is suitable for procedure sedation with a narrow therapeutic window, like fibreoptic intubation with spontaneous ventilation [12], [13], gastrointestinal upper endoscopic ultrasound (upper-GI EUS) [14] and endoscopic retrograde cholangiopancreatography (ERCP) [15]. Some studies reveal fewer interventions on the infusion device in TCI compared to manually-controlled infusion (MCI), making TCI easier to use [16]. A study with a small number of patients has described the application of TCI in FB sedation for patients under noninvasive ventilation support in an intensive care unit [17].

Thus, TCI is potentially feasible for FB sedation. However, as the optimal protocol has not yet been established, the present research designed three titration protocols to validate and establish the potential regiment of propofol TCI for FB sedation. The primary endpoint was safety, the proportion of patients with hypotension and hypoxemia, while the secondary endpoints were adjustment frequency, induction and recovery time, and propofol doses.

## Materials and Methods

This prospective, randomized study was conducted in the tertiary medical center Chang Gung Memorial Hospital. The study protocol was approved by the Chang Gung Medical Foundation Institutional Review Board (No. 98-3441A3). The protocol for this trial and supporting CONSORT checklist are available as supporting information; see Checklist S1 and Protocol S1. All of the enrolled patients provided written informed consent. Patients undergoing elective FB and sedation were screened for enrolment. The exclusion criteria were age <18 years, American Society of Anaesthesiologists (ASA) physical status classification IV or V, Mallampati score of 4, severe sleep apnoea syndrome (apnoea-hypopnea index >40), body mass index >42 in males or >35 in females, neurologic disorders or other conditions contributing to difficulty in assessing response, forced expiratory vital capacity (FVC) <15 ml/kg body weight, forced expiratory volume in one second (FEV1) <1000 ml, or FEV1/FVC <35%, chronic use of opioid drugs, and pregnancy. Patients with a known history of allergy to the study drugs, or to eggs, soybeans or sulfite products, were also excluded.

### Patient preparation

Blood pressure was monitored using an automated pressure cuff while heart rate and rhythm were monitored by three-lead electrocardiography. A peripheral pulse oximeter monitored the oxyhemoglobin saturation (SpO2) while a nasal cannula delivered oxygen 2 L/min. An intravenous catheter was placed in the forearm for drug administration. All parameters were monitored continuously except for blood pressure, which was recorded every 2 min.

An experienced bronchoscopist performed the FB via nasal route with assistance from a well-trained technician. Procedures of the FB were performed as described in previous studies [18], [19]. A well-trained staff responsible for sedation techniques monitored the cardio-pulmonary functions to determine the need for interventions, including increased oxygen delivery to 6L/min to maintain oxygen saturation above 90%, jaw support or manually assisted ventilation by ambubag for persistent desaturation to maintain adequate ventilation and airway patency, or fluid resuscitation and leg elevation for hypotension. All of the bronchoscopists and investigators were qualified for intensive and critical care and advanced cardiac life support. They were also familiar with the sedation drugs used for FB.

### Sedation protocol

The patient's weight, height, age, and gender were inputted into the TCI system (Injectomat TIVA Agilia, Fresenius Kabi, France) to calculate the infusion profile to achieve “target effect-site concentration” (Cet, the target setting of Ce) based on the Schnider model. The current Ce calculated by the model was displayed on the screen of the TCI pump and approached the desired Cet. Pre-medication was achieved by 2% xylocaine inhalation and 5 μg/kg alfentanil (1∶10 dilution) slow injection 1 min before induction [18], [19], [20].

The eligible enrolled patients were randomised by simple randomization according to a predetermined random computer code into three groups. These groups differed by level for titration, i.e., by Cet 0.5 μg/ml (Group 1), by Cet 0.2 μg/ml (Group 2), and by Cet 0.1 μg/ml (Group 3), during induction and procedure in order to maintain a stable sedation level and vital signs. The Cet of induction was set to 2 μg/ml initially. The Observer Assessment of Alertness and Sedation scale (OAA/S; 1, no response to shaking; 2, responds only to shaking; 3, responds only to name called loudly; 4, lethargic response to name called in normal tone; and 5, responds readily to name spoken in normal tone) [21], [22] was evaluated every 30 sec after the patients closed eyes during induction.

Upon reaching OAA/S 3∼2, the Ce level was recorded as induction Ce and set as the maintenance Cet during the procedures. The duration from the start of the propofol infusion to OAA/S 3∼2 was recorded as induction time. If OAA/S did not reach to 3 while Ce was 2 μg/ml, Cet was increased by the titration regimen every 90 sec until the OAA/S was 3∼2, at whitch time the Ce was set as the maintenance Cet. During maintenance, the Cet was increased by the titration regimen if the patient became irritable enough to interfere with the procedure or if there was persistent eye opening or talking. The Cet was reduced if the following adverse events occurred: SpO_2_ <90%, mean arterial blood pressure (MAP) <65 mmHg, or systolic blood pressure (SPB) <90 mmHg with any duration.

### Assessment

Data that were recorded from the beginning of induction to patient recovery in the bronchoscopy room included the proportion of patients with at least one event of hypotension and hypoxemia, frequency of Cet adjustment, Ce of induction and maintenance, propofol doses, and induction and recovery time.

### Sample size

A preliminary study was performed before this trial. Eleven, 13, and 12 patients underwent one of the three arms of TCI protocol, respectively, and were analysed. The proportion of patients in each of these groups with at least one episode of hypotension was 9.1%, 23.1%, and 8.3%, respectively. A difference of 14% was used to calculate the number of patient required to show the difference between the study groups titrated by Ce 0.5 and those by 0.2 μg/ml. The selected sample size was 98 for each group, considering 10% loss, for a total of 327 to yield 80% power for a two-sided test with a significance level of 5%.

### Statistics

Two populations were identified for the purposes of analysis. The intent-to-treat population (ITT) consisted of all randomized patients and the primary endpoint was analyzed in ITT. The secondary endpoints were analysed in patients receiving complete sedative intervention.

Data was expressed as number with percentage or mean with standard deviation. Normal distribution of continuous variables were tested by Kolmogorov-smirnov test and data were analysed by one-way ANOVA or Kruskall-Wallis test accordingly to evaluate difference between groups, while a Tukey post hoc test was performed if there was statistic significance of variables with in normal distribution. Patient characteristics and complications were analysed by Chi-square test or Fisher's exact test if sample size is small. A *p*<0.05 was considered statistically significant. An interim analysis was planned for the purpose of assessing safety. “An independent data and safety monitoring board periodically reviewed the efficacy and safety data.” Events with SpO2 less than 80%, lowest SBP less than 70% of baseline and mortality due to any cause were analyzed. The results of significance tests at the interim analyses were be considered significant if p<0.01. All of the statistical analyses were performed using the Statistical Package for Social Sciences version 13 (SPSS Inc., Chicago, IL, USA).

## Results

**Figure 1:**
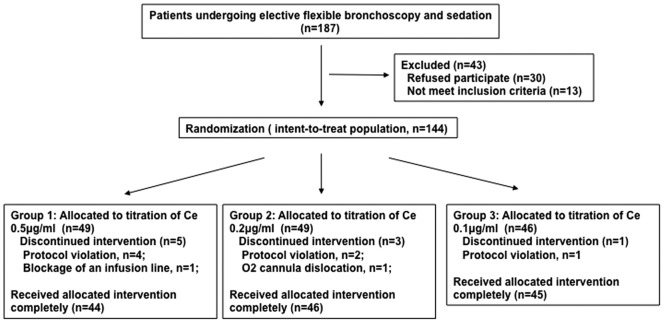
Patient disposition.

From February to August 2010, 144 patients undergoing elective FB were randomized (Figure 1). The study was closed early because of significantly severe hypoxemia events in Group 1 by interim analysis. Before closure, 44, 46, and 45 patients completed the intervention in the three groups, respectively. Each group had comparable baseline characteristics, indications for FB, and procedures performed in ITT (Table 1) and patients receiving complete intervention (data not shown). More than 80% of patients were outpatients and half received invasive biopsy.

**Table 1:** Patient characteristics, indications for flexible bronchoscopy, and procedures performed of the intent-to-treat group.

	Group 1 (n = 49)	Group 2 (n = 49)	Group 3 (n = 46)
**Patient characteristics**			
Age (SD), yr	61.6 (12.9)	63.3 (12.6)	64.1 (14.7)
ASA (range)	2 (1–3)	2 (1–3)	2 (1–3)
Male, n (%)	25 (51.0)	28 (57.1)	23 (50.0)
Weight (SD), Kg	58.3 (8.8)	60.3 (10.1)	59.4 (10.2)
BMI (SD)	22.8 (3.0)	23.3 (3.9)	23.1 (4.1)
Outpatient, n (%)	41 (83.7)	44 (89.8)	37 (80.4)
**Indications of FB, n (%)**			
Lung mass/nodule	29 (59.2)	33 (67.3)	28 (60.9)
Lung infiltration	12 (24.5)	11 (22.4)	13 (28.3)
Hemoptysis	2 (4.1)	2 (4.1)	3 (6.5)
Chronic cough	5 (10.2)	3 (6.1)	1 (2.2)
Other	1 (2.0)	0	1 (2.2)
**Procedures during FB, n (%)**			
Mini-probe EBUS	39 (79.6)	41 (83.7)	39 (84.8)
TBLB	22 (44.9)	27 (56.2)	22 (47.8)
Bronchial biopsy	6 (12.2)	6 (12.5)	7 (15.2)
Bronchial washing	33 (67.3)	30 (62.5)	31 (67.4)
Bronchial brushing	26 (53.1)	22 (45.8)	24 (52.2)
Bronchoalveolar lavage	11 (22.4)	9 (18.8)	9 (19.6)

Data are presented as mean ± standard deviation or number and percentage in parentheses. No statistically significant difference is found between groups.

Abbreviations: ASA, American Society of Anesthesiologists; BMI, body mass index; EBUS, endobronchial ultrasound; TBLB, trans-bronchial lung biopsy.

There was a significant difference in the proportion of patients with hypoxemia episodes in ITT (Table 2). The proportion of patients with SpO2 <70% was significantly higher in Group 1. These events mainly occurred during the procedures and were responsible for the early closure of this study. The proportion of patients with hypotension was not significantly different. All of the patients with hypoxemia or hypotension recovered spontaneously or after proper management. One patient in Group 1 and two in Group 3 developed pneumothorax and recovered completely after treatment.

**Table 2:** Proportions of hypoxemia and hypotension during bronchoscopic sedation of the intent-to-treat group#.

	Group 1 (n = 49)	Group 2 (n = 49)	Group 3 (n = 46)	p value
**Hypoxemia, n (%)**	33 (67.3)	23 (46.9)	19 (41.3)	0.03
Induction*	9 (18.4)	8 (16.3)	3 (6.5)	0.2
Procedure†	28 (57.1)	18 (36.7)	19 (41.3)	0.1
SpO_2_<80%	6 (12.2)	1 (2.0)	2 (4.3)	0.09
SpO_2_<70%	5 (10.2)	0	0	0.007
**Hypotension, n (%)**				
Induction*				
MAP<65 mmHg	0	1 (2.0)	0	0.4
SBP<90 mmHg	0	2 (4.1)	0	0.1
Procedure†				
MAP<65 mmHg	8 (16.3)	11 (22.4)	4 (8.7)	0.2
SBP<90 mmHg	7 (14.3)	7 (14.3)	5 (10.9)	0.9

Data are presented as number and percentage.

Abbreviations: SpO_2_: oxyhemoglobin saturation; MAP: mean arterial pressure; SBP: systolic blood pressure.

# The proportions of patients with at least one event of hypoxemia (SpO_2_<90%) or hypotension (MAP<65mmHg or SBP<90mmHg) during the entire procedure.

* From alfentanil administration to Observer Assessment of Alertness and Sedation scale less than 3.

† From insertion of bronchoscope to its removal.

**Figure 2:**
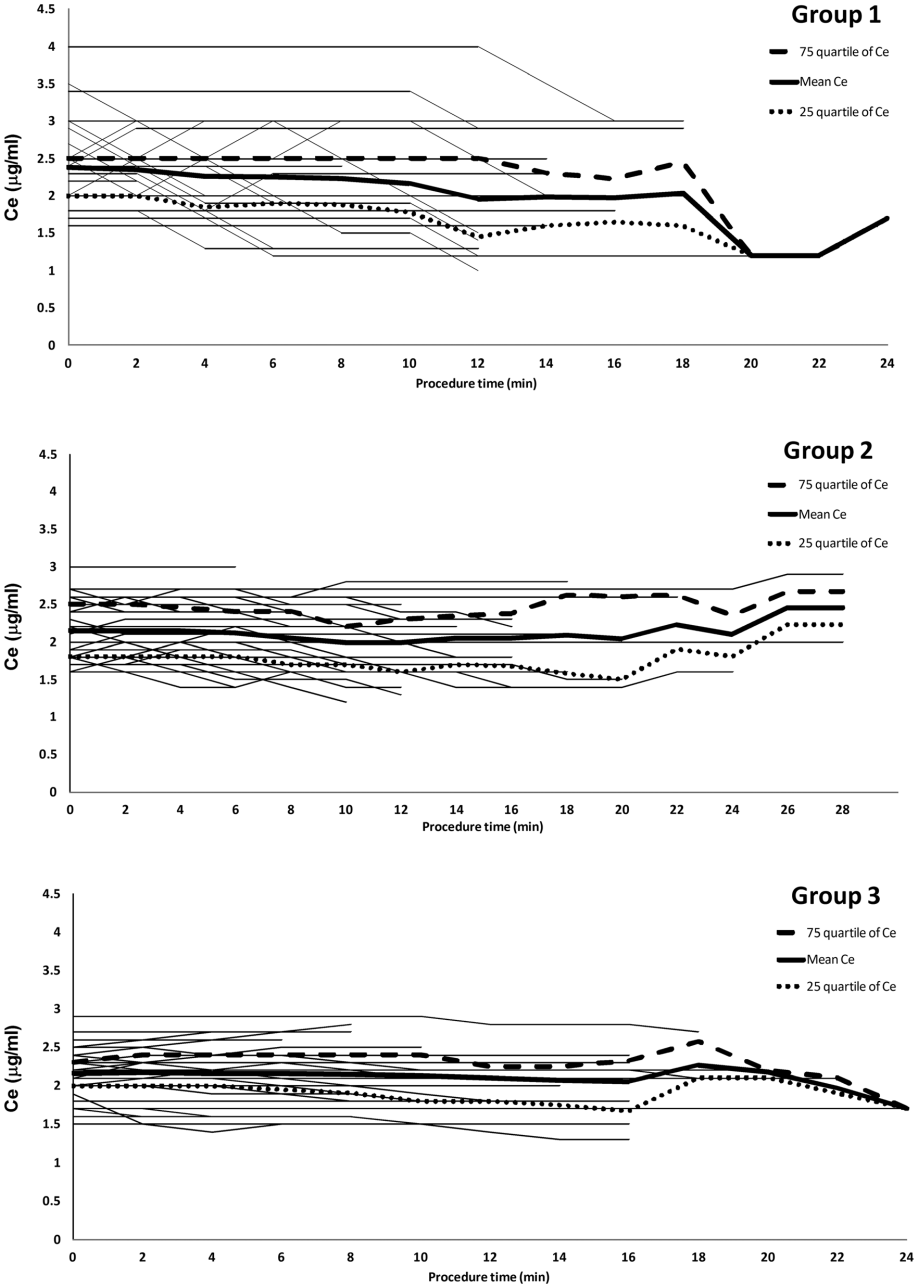
Maintenance effect-site concentration (Ce) of the three groups during bronchoscopy. Groups 1, 2, and 3 of Cet 0.5, 0.2, and 0.1 μg/ml titration, respectively, maintained steady sedative level and vital signs during bronchoscopy. The maintenance Ce was recorded after induction to the end of bronchoscopy.

The sedation outcome during induction and the procedure showed that the induction doses and duration were similar in each group of patients receiving complete intervention, but the induction Ce differed significantly between groups (Table 3). There was a trend of higher adjustment frequency during induction in Group 3 but it was not statistically significant. The total propofol doses and the procedure and recovery time were similar in each group. The mean maintenance Ce was significantly higher in Group 1. In terms of maintenance Ce level throughout the FB, the initial maintenance Ce was higher in Group 1 and gradually titrated to lower levels (Figure 2). The maintenance Ce in Groups 2 and 3 were relatively smooth during FB.

**Table 3:** Bronchoscopy and sedative outcomes of the patients receiving complete intervention.

	Group 1 (n = 44)	Group 2 (n = 46)	Group 3 (n = 45)	p value
**Induction^#^**				
Doses of A, μg	293.4 (60.7)	310.7 (54.2)	308.9 (64.5)	0.7
Dose of P, mg	55.4 (21.2)	49.6 (18.7)	53.2 (27.2)	0.7
Induction Ce^#^, ug/mg	2.4 (0.5)	2.1 (0.4)	2.1 (0.3)	0.005
Induction time, sec	235.1 (118.0)	212.6 (117.6)	262.0 (20 0.1)	0.7
**Procedures** *				
Procedure time, min	18.2 (8.3)	18.8 (7.5)	18.0 (8.5)	0.7
Doses of P, mg	147.3 (56.2)	147.2 (65.6)	151.6 (65.9)	0.8
Maintenance Ce	2.4 (0.5)^& ⊕^	2.1 (0.4)^ &^	2.2 (0.3) ^⊕^	0.006
Time to orientation†, min	13.7 (10.4)	15.2 (12.6)	12.9 (9.5)	0.6
**Frequency of Cet adjustment** ¶	2 (0∼6)	3 (0∼6)	3 (0∼11)	0.3
Induction	1 (0∼4)	1 (0∼5)	1 (0∼9)	0.4
Procedure	1 (0∼5)	1 (0∼5)	1 (0∼6)	1.0

Data are presented by mean (standard deviation) unless otherwise indicated. Abbreviations: A, alfentanil; P, propofol; Ce: effect-site concentration; Cet: target setting of Ce.

#From starting propofol infusion to Observer Assessment of Alertness and Sedation scale 3∼2.

* From insertion of bronchoscope to its removal.

† From bronchoscope removal to patients could open eyes spontaneously, correctly recall date of birth, and perform finger-nose test.

Turkey post hot test. & p = 0.007; ⊕ p = 0.034.

¶ Data is presented by median (range).

We found that there were 14% of the patients who required no more Cet adjustment during all the procedures. Comparing the patients requiring at least once of Cet adjustment during sedation with those who did not, there was no difference in patient characteristics, but there was a faster induction with smaller propofol doses for induction and overall procedures in the patients requiring no adjustment (Table 4). The procedure time and procedures performed (data not shown) were similar between the two groups except more TBLB were performed in the patients requiring no adjustment (78.9 vs. 47.0%, p = 0.012). Fewer complications occurred in the patients requiring no adjustment and the three episodes of hypoxemia occurred during induction.

**Table 4:** Subgroup analysis of patients requiring no adjustment or at least one adjustment of Cet during whole procedures.

	Frequency of Cet adjustment = 0 (n = 19)	Frequency of Cet adjustment>0 (n = 116)	p value
**Patient characteristics**			
Age (SD), yr	66.1 (14.9)	62.7 (13.2)	0.3
ASA (range)	2 (1–3)	2 (1–3)	0.9
Male, n (%)	11 (57.9)	63 (54.3)	0.8
Weight (SD), Kg	59.4 (7.9)	59.6 (10.1)	0.9
BMI (SD)	23.2 (3.4)	23.1 (3.7)	0.9
Outpatient, n (%)	14 (73.7)	100 (86.2)	0.2
**Sedative outcomes**			
Induction propofol dose, mg	32.5 (4.1)	56.8 (22.7)	<0.001
Induction Ce, ug/mg	1.76 (0.17)	2.28 (0.41)	<0.001
Induction time, sec	87.6 (34.9)	266.9 (147.9)	<0.001
Total propofol doses, mg	106.3 (37.4)	155.7 (63.0)	<0.001
Procedure time, min	18.0 (8.1)	18.4 (8.1)	0.8
Hypoxemia at least once	3 (15.8)	66 (56.9)	0.001
Hypotension at least once	0 (0)	28 (24.1)	0.013

Data are presented as mean ± standard deviation or number and percentage in parentheses.

Abbreviations: ASA, American Society of Anesthesiologists; BMI, body mass index; Ce: effect-site concentration; Cet: target setting of Ce.

## Discussion

To date, this is the first prospective randomized control trial of TCI with propofol for FB sedation. Although the study was closed early because of safety concerns, valuable information was nonetheless obtained. TCI with 0.5 μg/ml Ce titration contributed to significant hypoxemia during FB and can not be recommended. The relatively high adjustment frequency of TCI with 0.1 μg/ml Ce titration offset the advantages of TCI. Lastly, TCI with propofol titrated by 0.2 μg/ml was the possible potential regimen for FB sedation. However, further study is required to draw definite conclusions about 0.2 and 0.1 μg/ml titration because of the low power rating of the present study.

The three compartment model of propofol has been integrated into the plasma-targeted Marsh model for better PK control to improve the efficacy of sedation and anaesthesia procedures [9]. Because of the phenomenon of hysteresis, i.e., the lag between plasma concentration and brain effect [23], an artificial effect site compartment has been created to improve propofol PK in the modified Marsh model [10] and Schnider model [6], [11] in the open TCI system, which could directly titrate the propofol concentration in the brain. Compared with the Marsh plasma-targeted model, the Schnider model further includes the variables of patients' age and height to estimate the compartment volume and the equilibrium rate constants. Base on these accuracy conferring variables, the Schnider model is used for FB sedation in the present study.

Induction Cet was targeted at 2.0 μg/ml in the present study according to our clinical experience and relevant studies. Janzen et al. reported that 2.1 μg/ml was the propofol target concentration for 50% of their patients to attain a sedation level of response to physical stimulation [24]. Xu et al. observed that Ce 2.2 μg/ml of propofol caused a loss of response in 50% of their Chinese patients [25]. In the present study, the mean Ce for induction was 2.1 μg/ml in both Groups 2 and 3. The higher induction Ce in Group 1 was possibly due to the higher overshoot infusion profile, which attempts to create a plasma concentration gradient to achieve the higher desired target effect site concentration. A higher induction Ce contributes to the further higher maintenance of Ce, which was reduced due to unstable vital signs during the latter course of FB (Table 2 and Figure 2). This can partially explain why Group 1 experienced more complications.

Compared to Group 1's, the maintenance Ce was relative steady in the other 2 groups at around 2.1 to 2.2 μg/ml with deviation 0.3∼0.4, which is a valuable reference for TCI application for FB sedation (Figure 2). Although there is no statistical difference in adjustment frequency between Groups 2 and 3, there was a trend of more intervention during induction (1.0±1.0 vs. 1.2±1.4 vs. 1.8±2.1, respectively) with a wider adjustment range throughout FB in Group 3, which may offset the advantages of TCI. Recently, Clouzeau et al. demonstrated the modest hemodynamic change and good patient tolerance of TCI for bronchoalveolar lavage (BAL) during FB in 23 patients with respiratory failure under noninvasive ventilation support [17]. The initial target was set at 0.6 μg/ml for lighter sedation level (OAA/S 4∼3) with a titration of 0.2 μg/ml which is comparable with our conclusion. The mean Ce was lower (1.49±0.46 μg/ml) due to the desired lower sedative level and the simply performed BAL with a shorter procedure time. The FB procedures performed in the present randomized controlled study, however, were more complicated with longer duration under deeper sedation level without ventilator support.

It has been proven that patients undergoing FB experience procedure-related symptoms and the academic guidelines recommend providing sedation to all patients undergoing FB, except when there are contraindications [4], [26]. Although administration of short-acting opioids alone provides pain control and antitussive effect during FB [27], lack of an amnesic effect can be stressful for anxious patients undergoing interventional FB procedures [28]. Midazolam provides effective FB sedation but it may lead to delayed recovery [3], [29]. Other studies reveal that bolus propofol provides better recovery profile without an increase in hemodynamic complications [3]. Nonetheless, controversy about propofol bolus administration at the physician's discretion is persistent due to individual PK differences with the associated risk of fluctuating sedative levels that could lead to cardiopulmonary depression [5], [6]. The predictive performance of TCI, on the other hand, has been validated and proven clinically acceptable [30], but evidence for TCI application in FB sedation has still not been established. The safety and sedative profiles in the present randomized-controlled study can provide information to evaluate the application of TCI in FB sedation. The average portion of hypoxemia events of the three study groups was about 50% (Table 2), which is higher than the reports employing MCI of propofol (Stolz et al.: 32% [3] and Lo at al.: 39% [19]), the report with patient-controlled sedation (Yoon et al.: 12.5% [31]) and nurse-administrated sedation for FB (Bosslet et al.: 3.8% [32]). From the aspect of non-FB procedures, Fanti et al. [14] reported the plasma-target propofol TCI sedation in patients undergoing upper-GI EUS resulted no hypoxemia events: the induction concentration was 4 mg/ml with 0.5 mg/ml titration and there was higher oxygen support (40%) than in the present study. The authors also reported a similar regimen for ERCP, with additional fentanyl bolus as needed [15]. Even though the incidence of SpO2 less than 85% was 1.9%, these non-FB procedures cause less airway compromise and less irritant cough than FB. Despite the differences in oxygenation support, desired sedative level and sedative protocol between these studies, something else is also relevant to the safety of TCI. Whereas further analysis revealed a similar hypoxemic rate as MCI's during procedures for titration at 0.2 μg/ml (37%, Table 2), the hypoxemic rate was relatively high during induction. In the induction profile, for example, the doses and timing of opioid administration during induction and the level of induction Ce required further investigation to improve safety during induction. Alfentanil is ideal for FB because of its fast onset and short duration [33], [34], [35]. The aim of alfentanil is to provide antitussive effects and may modify the pharmacokinetics of propofol, which reduces the required propofol dose [36], [37]. Dosage of alfentanil in the studies about premedication for TCI in bronchoscopy ranges from 4–5 to 10 μg/kg [13], [19], [38]. In the work about different dosages of alfentanil with propofol for laryngeal mask insertion performed by Yu et al., 5 and 10 μg/kg of alfentanil provided good effect to reduced cough and gagging reflex but 5 μg/kg of alfentanil caused no prolong apnea [20]. Based on these evidences, we use 5 μg/kg of alfentanil as our premedication. Reducing or even managing without opioid administration may reduce the rate of hypoxemia but the effect on propofol doses and its resulting severity of cough requires further research. Moreover, studies about the performance error of current PK models of propofol point to a lag between Ce and exact consciousness level. These models, including the Schnider model, tend to underestimate the exact plasma propofol concentration at the early phase (the first 21 min) of operation [30], [39]. It is unclear that this is the case during FB. Adding an objective monitor, like Bispectral index (BIS), during TCI for FB sedation may be a way of clarifying this issue. It has been reported that the incidence of hypoxemia was 35% and 39% respectively for the BIS-guided propofol bolus and continuous infusion in FB sedation [19], [40], which implies the potential advantage of pharmacodynamic monitoring in FB sedation. The cost-effectiveness of pharmocodynamic monitoring by BIS and PK controlled by TCI, however, warrants further investigation.

After setting TCI according to the individual information of patients, 14% of the patients required no more adjustment the Cet because of the steady sedative level and vital signs. Subgroup analysis revealed there was no difference in basic characteristics but the induction time was faster with smaller propofol doses in the patients requiring no adjustment. The procedure time and procedures performed were similar in the two groups and overall propofol doses were also smaller in the patients requiring no adjustment. This implies such patients are more sensitive to propofol and the TCI program can provide a better fit for the PK model better in these patients. Interpatient susceptibilities to propofol has been described as the function of the differences in cardiac output, hepatic perfusion, and body fat, as well as due to the haplotype differences in metabolic genes [41]. This issue needs additional research to provide more information to help improve TCI program designs and patient selection for propofol sedation.

The present study has certain limitations. First, its power is not adequate because of the early closure. Even so, the information provided is valuable in clinical practice and can inspire future studies to ameliorate TCI application on FB sedation. Second, the investigators who responded to the sedation procedures were not blinded to the patients' titration regimen. For the safety reason, investigators inputted and confirmed the correct titration regimen on the screen of the infusion pump. Nonetheless, the primary endpoints were hypoxemia and hypotension events, which were recorded objectively.

In conclusion, the TCI of propofol titration at 0.5 μg/ml is risky, particularly for hypoxemia. A subgroup of patients required no more TCI adjustment during whole procedure with fewer complications. Additional research is needed to draw conclusions about the feasibility of 0.2 and 0.1 μg/ml titration and opioid administration during induction.

## Supporting Information

Figure S1 **CONSORT 2010 Flow Diagram.**
(DOC)

Checklist S1 **CONSORT Checklist.**
(DOC)

Protocol S1 **Trial protocol.**
(DOC)

Protocol S2 **Trial protocol (Chinese version).**
(DOC)

Text S1 **Statement of IRB.**
(TIFF)
