# Supplementary material for: The Potential Regimen of Target-Controlled Infusion of Propofol in Flexible Bronchoscopy Sedation: A Randomized Controlled Trial
Source: PLoS One. 2013 Apr 24;8(4):e62744. doi: 10.1371/journal.pone.0062744 (PMC3634750; doi:10.1371/journal.pone.0062744)
Supplement: Figure S1 — CONSORT 2010 Flow Diagram. (DOC) [file pone.0062744.s001.doc]

**CONSORT 2010 Flow Diagram**

**Allocation**

**Analysis**

**Follow-Up**

**Enrollment**

Assessed for eligibility (n= 187)

Excluded (n= 43 )

♦  Not meeting inclusion criteria (n=13 )

♦  Declined to participate (n=30 )

♦  Other reasons (n=0 )

Lost to follow-up (give reasons) (n= 0 )

Discontinued intervention (give reasons) (n= 0 )

Allocated to Group 3, n=46

♦ Received allocated intervention, n=45

♦ Did not receive allocated intervention

Protocol violation n=1

Analysed (n= 45)
♦ Excluded from analysis (give reasons) (n=0 )

Randomized (n= 144 )

Lost to follow-up (give reasons) (n=0 )

Discontinued intervention (give reasons) (n= 0 )

Allocated to Group 1, n=49

♦ Received allocated intervention, n=44

♦ Did not receive allocated intervention

Protocol violation n=4

Blockage of an infusion line n=1

Analysed (n= 44 )
♦ Excluded from analysis (give reasons) (n=0 )

Lost to follow-up (give reasons) (n= 0 )

Discontinued intervention (give reasons) (n= 0 )

Allocated to Group 2, n=49

♦ Received allocated intervention, n= 46

♦ Did not receive allocated intervention

Protocol violation n=2

O2 cannula dislocation n=1

Analysed (n= 46 )
♦ Excluded from analysis (give reasons) (n= 0 )
