# Supplementary material for: The Potential Regimen of Target-Controlled Infusion of Propofol in Flexible Bronchoscopy Sedation: A Randomized Controlled Trial
Source: PLoS One. 2013 Apr 24;8(4):e62744. doi: 10.1371/journal.pone.0062744 (PMC3634750; doi:10.1371/journal.pone.0062744)
Supplement: Protocol S1 — Trial protocol. (DOC) [file pone.0062744.s003.doc]

**Study protocol**

1. Title

| Target-Controlled infusion of propofol for flexible bronchoscopy sedation: Determining the optimal titrating regimen. |
| --- |

1. Background and Aims

| Propofol is ideal for bronchoscopy sedation because of its fast onset and quick recovery effect. Our research and reports from different investigators demonstrate that patients received propofol sedation recover fast with excellent satisfaction for bronchoscopy. However, the amount of propofol for induction and maintenance is calculated simply by patient’s body weight and physicians’ experience. For those non-anesthesiologists, who perform sedative work outside the operating room, and inexperienced anesthesiologist without fully considering the individual pharmacokinetic and pharmacodynamic differences may generate unstable drug plasma concentration and increase cardio-respiration suppression. Therefore, a manner which can assess and measure objectively individual pharmacokinetic differences may improve the sedative quality and decrease the complication rate.  A model called “Target-controlled infusion, TCI”, built from massive pharmacokinetic samples of propofol, could now give precise pharmacokinetic control. Several pharmacokinetic models built-in in TCI, includes the Schnider model which use concentration of effect site (Ce, the propofol concentration in the brain) as the sedative guide. The model integrates individual variants of age, height, weight and gender to calculate the infusion profile to achieve predetermined steady Ce. Because of the unique consideration of individual pharmacokinetic variants and Ce targeting, TCI provides predictable sedative level and is suitable for procedures requiring narrowing therapeutic level. Beside general anesthesia, TIC has been applied in breast biopsy, upper gastrointestinal endoscopic untrasound and endoscopic retrograde cholangiopancreatography at outpatient clinic. According to these evidences, there is potential role of TCI in bronchoscopic sedation.  Based on current evidence and our experience, we design this study to evaluate the optimal regimen for induction and procedure during bronchoscopy. We hope this study could provide the more safety and efficient bronchoscopic sedation for patients and physicians. |
| --- |

1. Study design

| **Duration:** 2009/12 to 2012/12  Subjects: patients required elective bronchoscopy and bronchoscopic sedation.  Patient number: 327.  **Patient distribution**: Initial Ce of induction is 2μg/ml. Eligible patients are random into three groups:  Group 1: Titration by 0.5μg/ml during induction and procedure；  Group 2: Titration by 0.2μg/ml during induction and procedure；  Group 3: Titration by 0.1μg/ml during induction and procedure,  to maintain stable sedative and vital signs level.  **Evaluation and statistics**  Primary outcomes:   1. The frequency of Ce adjustment, mean Ce and safety during induction. 2. The frequency of Ce adjustment, mean Ce and safety during procedure. 3. Induction time, and immediate and later recovery time.   Secondary outcomes:   1. The satisfaction about bronchoscopy. 2. The total drug doses during induction and procedure. 3. The cooperation of patients from the view of bronchoscopists.   **Data analysis**   1. Basic characteristics of patients. 2. Mean Ce, doses and time for induction, frequency of Ce adjustment and complication. 3. Mean Ce, doses and time for procedure, frequency of Ce adjustment, complication and recovery time. 4. Frequency of patient self-report symptoms and rank of satisfaction about bronchoscopy. 5. The rank of cooperation of patients from the view of bronchoscopists   Age, Ce, doses, induction time, procedure time and recovery time are expressed as mean with standard deviation. Adjustment and verbal analogous scale are expressed as median with range. Age, weight, cardiopulmonary parameters, induction, procedure and recovery time are analyzed by Student’s t-test. Ce, doses, frequency of adjustment and verbal analogous scale are analyzed by Mann–Whitney U test. Characteristics of patients, frequency of bronchoscopic related symptoms and complication are analyzed by Chi-square test. P value less 0.05 means significance. |
| --- |

計畫主持人簽名：林定佑 　日期：ˍˍˍˍˍ

協同主持人簽名：郭漢彬 　日期：ˍˍˍˍˍ

協同主持人簽名：羅友倫 　日期：ˍˍˍˍˍ
