# Supplementary material for: The Potential Regimen of Target-Controlled Infusion of Propofol in Flexible Bronchoscopy Sedation: A Randomized Controlled Trial
Source: PLoS One. 2013 Apr 24;8(4):e62744. doi: 10.1371/journal.pone.0062744 (PMC3634750; doi:10.1371/journal.pone.0062744)
Supplement: Protocol S2 — Trial protocol (Chinese version). (DOC) [file pone.0062744.s004.doc]

**中文計畫摘要**

1. 試驗主題

| 標靶控制普帕芙輸液應用於軟式支氣管鏡鎮靜術: 最適滴定劑量的決定。 |
| --- |

1. 背景及研究目的

| 普帕芙(Propofol)具有較快且較短的作用時間，因此是理想的氣管鏡鎮靜用藥。相關文獻以及我們的經驗中，接受普帕芙鎮靜的病人術後可快速的恢復，並表現對檢查高度的滿意度。然而，傳統計算普帕芙輸注劑量是單純根據體重，並沒有考慮到病人個別的藥物動力學因素，且調整多依照醫師的個人經驗，因此可能會造成不穩定的藥物血中濃度 (plasma concentration, Cp)。因此，以客觀的依據來設定與調整普帕芙的輸注劑量是有需要的。  現在經由標靶控制輸注系統 (Target-controlled infusion, TCI)已可達到精確的藥物動力學控制。依據大量的普帕芙藥物動力學樣本所建立的TCI，內建有以作用位置濃度(effect site, Ce，即腦中普帕芙濃度)為目標的模組。此模組納入了年齡、身高、性別和體重的變數，電腦可運算出達到穩定Ce的流速計畫。此模組考慮到病人的個別差異，且直接調控腦中普帕芙濃度，因此它提供了可預期的安全鎮靜深度，相當合適於需要維持狹窄治療濃度的處置。除了全身麻醉外，TCI已應用於如門診乳房切片檢查，上消化道內視鏡超音波和內視鏡逆行性膽胰造影術的鎮靜。基於以上特性，我們認為TCI有潛力提供更有效率的氣管鏡鎮靜術。  我們參考TCI的文獻以及我們的臨床經驗，設計此研究計畫來探討TCI應用於軟式支氣管鏡鎮靜術時，誘導鎮靜和術中維持鎮靜最有效與安全的滴定劑量。希望藉由這個研究，能提供有效安全的TCI操作來應用於氣管鏡鎮靜術，並提供相關研究者，對於氣管鏡鎮靜術有更進一步的了解。 |
| --- |

1. 試驗設計

| **執行期間:** 2009年12月起2012年12月。  **受試者**: 選擇性接受軟式支氣管鏡以及氣管鏡鎮靜術的病人。  由研究者於支氣管檢查室，對於符合條件(見試驗計畫書)的病人解釋鎮靜術的做法並回答患者所關心的問題以及知情同意書的內容。  受試人數:327人  **研究方法**: 誘導鎮靜目標濃度為2μg/ml。病人隨機分為三組: 第一組在誘導鎮靜和術中以0.5μg/ml上下調整；第二組在誘導鎮靜和術中以0.2μg/ml上下調整調整; 第三組在誘導鎮靜和術中以0.1μg/ml上下調整，以維持穩定的鎮靜深度(意識鎮靜)和生命徵象。  **評估及統計方法**  **主要結果**： 1. 誘導鎮靜的調整次數、平均Ce和安全性。 2. 術中維持鎮靜的調整次數、平均Ce和安全性。3. 誘導鎮靜的時間和恢復時間。  **次要結果：**1. 患者對於氣管鏡術的滿意度。 2. 誘導鎮靜和術中使用普帕芙的劑量。 3. 氣管鏡操作者評估患者整體的配合度。  **數據分析**:   1. 病患基本資料。 2. 達到誘導鎮靜的Ce, 調整濃度次數，所需的時間和劑量，併發症的發生。 3. 術中維持鎮靜的平均Ce，調整濃度次數和原因，藥物的劑量，術中併發症,恢復時間的長短。 4. 病人關於自我呈述的症狀，對於氣管鏡術的滿意度。 5. 氣管鏡操作者對於病人配合的滿意度。   病患的年齡，達到誘導鎮靜的Ce，藥物的劑量，誘導鎮靜時間，氣管鏡術的時間及檢查後恢復時間以平均值和標準偏差呈現。調整濃度次數和問卷中的verbal analogous scale以中位數與範圍呈現。兩組之間的年齡，體重，心肺參數、鎮靜時間和恢復時間使用Student’s t-test分析。Ce、藥物劑量、調整濃度次數和Verbal analogous scale使用Mann–Whitney U test分析。對於患者的特性，氣管鏡相關症狀，併發症的發生與否，使用Chi-square test分析。以p值< 0.05 為明顯的統計意義。 |
| --- |

計畫主持人簽名：林定佑 　日期：ˍˍˍˍˍ

協同主持人簽名：郭漢彬 　日期：ˍˍˍˍˍ

協同主持人簽名：羅友倫 　日期：ˍˍˍˍˍ
