# Supplementary figures and images for: The Potential Regimen of Target-Controlled Infusion of Propofol in Flexible Bronchoscopy Sedation: A Randomized Controlled Trial
Source: PLoS One. 2013 Apr 24;8(4):e62744. doi: 10.1371/journal.pone.0062744 (PMC3634750; doi:10.1371/journal.pone.0062744)

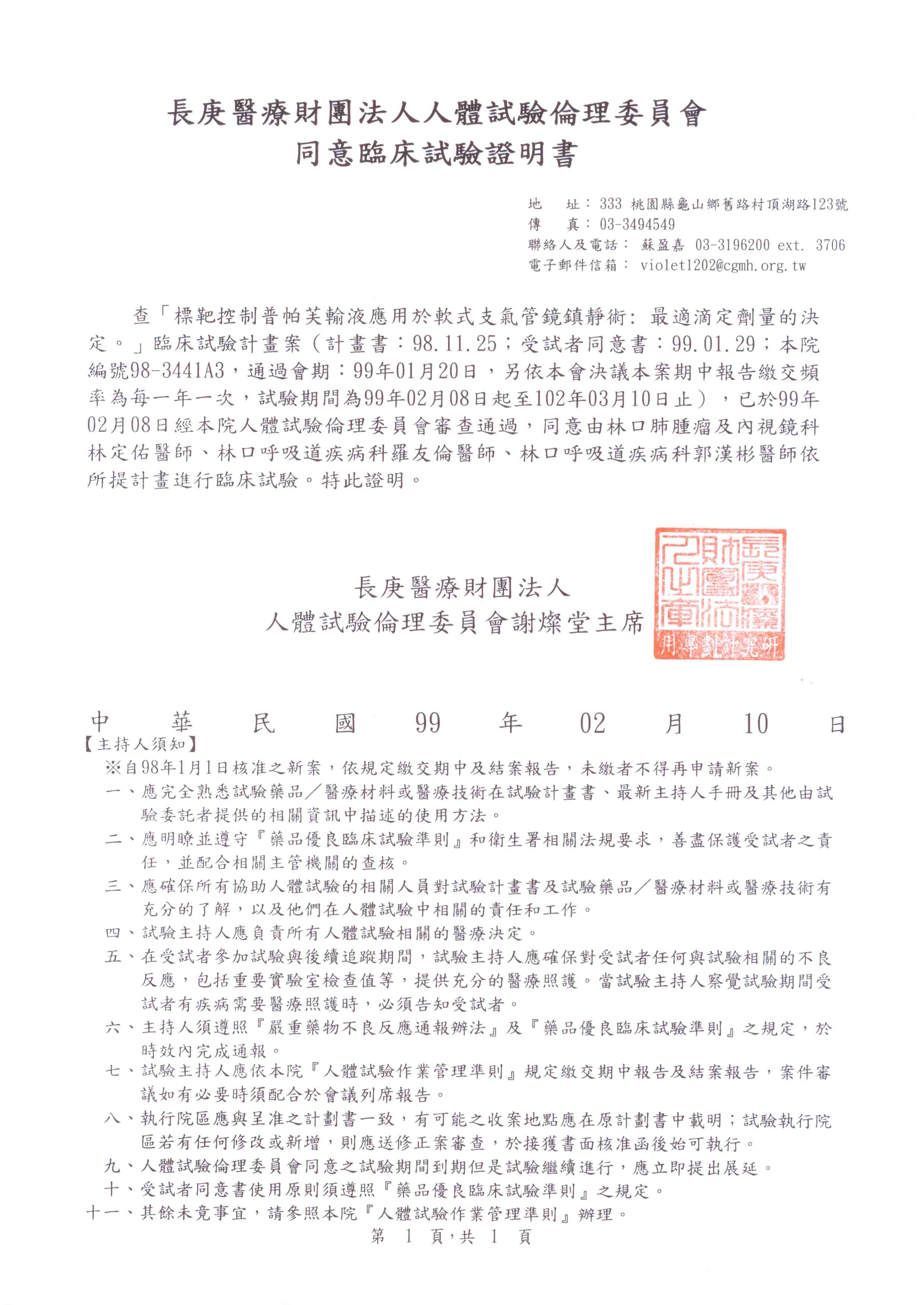

Supplement: Text S1 — Statement of IRB. (TIFF) [file pone.0062744.s005.tif]
